# Outcomes and risk factors for cancer patients undergoing endoscopic intervention of malignant biliary obstruction

**DOI:** 10.1186/s12876-015-0399-7

**Published:** 2015-12-04

**Authors:** Georg-Martin Haag, Thomas Herrmann, Dirk Jaeger, Wolfgang Stremmel, Peter Schemmer, Peter Sauer, Daniel Nils Gotthardt

**Affiliations:** Department of Medical Oncology, National Center for Tumor Diseases, University Hospital Heidelberg, Im Neuenheimer Feld 460, Heidelberg, 69120 Germany; Department of Gastroenterology, Toxicology and Infectious Diseases, University Hospital Heidelberg, Im Neuenheimer Feld 410, Heidelberg, 69120 Germany; Department of Internal Medicine I, Gastroenterology, Hematology and Medical Oncology, Hospital Heide, Heide, 25746 Germany; Department of Surgery, University Hospital Heidelberg, Im Neuenheimer Feld 110, Heidelberg, 69120 Germany; Liver Cancer Center Heidelberg (LCCH), University Hospital Heidelberg, Im Neuenheimer Feld 460, Heidelberg, 69120 Germany

**Keywords:** Cancer, Pancreatic cancer, Biliary obstruction, Cholangitis, Chemotherapy, Systemic therapy

## Abstract

**Background:**

Malignant bile duct obstruction is a common problem among cancer patients with hepatic or lymphatic metastases. Endoscopic retrograde cholangiography (ERC) with the placement of a stent is the method of choice to improve biliary flow. Only little data exist concerning the outcome of patients with malignant biliary obstruction in relationship to microbial isolates from bile.

**Methods:**

Bile samples were taken during the ERC procedure in tumor patients with biliary obstruction. Clinical data including laboratory values, tumor-specific treatment and outcome data were prospectively collected.

**Results:**

206 ERC interventions in 163 patients were recorded. In 43 % of the patients, systemic treatment was (re-) initiated after successful biliary drainage. A variety of bacteria and fungi was detected in the bile samples. One-year survival was significantly worse in patients from whom multiresistant pathogens were isolated than in patients, in whom other species were detected. Increased levels of inflammatory markers were associated with a poor one-year survival. The negative impact of these two factors was confirmed in multivariate analysis.

In patients with pancreatic cancer, univariate analysis showed a negative impact on one-year survival in case of detection of *Candida* species in the bile. Multivariate analysis confirmed the negative prognostic impact of *Candida* in the bile in pancreatic cancer patients.

**Conclusion:**

Outcome in tumor patients with malignant bile obstruction is associated with the type of microbial biliary colonization. The proof of multiresistant pathogens or *Candida*, as well as the level of inflammation markers, have an impact on the prognosis of the underlying tumor disease.

## Background

Malignant biliary obstruction is a frequent complication in patients with advanced tumor diseases. Both central hepatic metastases and hilar lymph node metastases of gastrointestinal and non-gastrointestinal cancers, but also tumors of the pancreatic head often result in a biliary compression.

Obstructive jaundice is associated with increased morbidity and mortality. Furthermore, intact bilirubin excretion is mandatory for the administration of many chemotherapeutical and targeted agents.

Only limited data exist concerning the role of jaundice in patients with metastatic disease. In a retrospective study published in 1994, median survival in patients with jaundice due to metastatic colorectal cancer was approximately 3–5 months [1]. However, taking into consideration the increasing number of therapeutic agents available for the treatment of metastatic colorectal cancer, more therapeutic options are available after improvement of biliary drainage, resulting in prolonged survival in patients. Furthermore, in tumors being considered as chemorefractory such as hepatocellular carcinoma (HCC), normalization of cholestasis parameters is mandatory to initiate systemic targeted treatment, such as sorafenib [2].

In patients with pancreatic carcinoma, biliary obstruction occurs in approximately 70–90 % of all cases, possibly leading to a deterioration of the patients’ general condition due to cholangitis, malabsorption or liver failure [3]. Although surgical bypass has demonstrated a low rate of recurrent jaundice, the surgical procedure itself carries a significant risk of perioperative morbidity and mortality [4].

Endoscopic retrograde cholangiopancreatography (ERCP) has been the preferred method for patients with malignant biliary obstruction since the late 1980s. Studies showed an equivalent success rate and decreased morbidity and mortality for this method in comparison with percutaneous or surgical approaches [5–7]. Yet, in patients with resectable pancreatic cancer, immediate resection of the tumor without a previous ERC is the preferred option [8].

Placement of a plastic or metal stent has been shown to be an effective method in improving biliary flow. Common complications include cholangitis and stent occlusion, whereas pancreatitis is only rarely observed post-ERCP [3]. The optimal type of stent (plastic or metal) is still a matter of debate. Plastic stents have been used as a standard method for biliary drainage; however, regular stent exchange is necessary every 3–4 months in order to prevent stent obstruction due to biliary sludge. Thus, metal stents are preferred in patients with an expected survival of more than 3 to 4 months [9].

Development of a biofilm is a critical event in the pathogenesis of cholangitis and the blockade of stents. Effectiveness of antibiotic treatment is limited against bacterial colonization of the biofilm [10].

The impact on biliary bacterial or mycotic infection in tumor patients with malignant biliary obstruction on survival is poorly studied. Data from patients undergoing liver transplantation show a negative prognostic impact on the proof of enteric bacteria or *Candida* species regarding time till organ failure [11–13].

In this prospective, observational study we aimed to identify the spectrum of bactobilia and fungobilia in patients with malignant obstruction, and to explore the association of this spectrum with clinical outcome.

## Patients and methods

The prospective, observational study was conducted at the Center for Endoscopy of the University Hospital Heidelberg in collaboration with the Department of Gastroenterology and the Department of Medical Oncology at the National Center for Tumor Diseases, Heidelberg. Patients were recruited consecutively. Data of patients undergoing ERC for malignant biliary obstruction were recorded in a prospective database. Biliary drainage was performed whenever technically possible.

Bile samples were obtained after selective intubation before any therapeutic procedure was performed. When bile could not be aspirated directly after cannulation, a small amount of sterile saline (2 – 4 ml) was applied and aspiration was reattempted. Aliquots of all biliary samples were placed in a sterile glass tube containing medium for anaerobic and aerobic bacterial cultures. The material was delivered to the microbiology laboratory within 2 h of collection and cultured aerobically and anaerobically according to standard laboratory protocols.

The underlying tumor disease including the stage of the disease, the proof of bacteria or fungi, as well as laboratory values including bilirubin and C-reactive protein (CRP) at the time of intervention were recorded. The previous or subsequent application of systemic therapy (e.g. chemotherapy) was documented.

Assuming that the biliary infection would rather influence the short-term outcome whereas the long-term outcome is determined by the underlying tumor disease, patients’ outcome was followed for a maximum of one year, patients lost to follow-up were censored at the time of the last documented contact. All patients provided informed consent, the study was performed in accordance with the Declaration of Helsinki. The study was approved by the ethical committee of the University of Heidelberg.

ERCP was performed in an inpatient setting. Concurrent antibiotic treatment or peri-interventional prophylaxis were performed at the discretion of the responsible physician.

### Statistical analysis

Continuous data were compared using the nonparametric Mann–Whitney *U* test. Frequency differences were compared using the chi-squared test or Fisher’s exact test where appropriate. The actuarial survival rate was estimated using the Kaplan-Meier product limit estimator. Differences between the actuarial estimates were tested using the log rank test. Cox regression analysis was performed for multivariate analysis. Differences were considered significant if p was <0.05. All analyses were performed using PASW Statistics 21.0 (SPSS Inc., Chicago, IL).

## Results

### Patient characteristics

Between October 2006 and December 2008, a total of 163 patients with advanced cancer diseases undergoing ERC were recorded.

The most frequent tumors were advanced pancreatic carcinoma (37 %), followed by cholangiocarcinoma (21 %) and metastatic colorectal carcinoma (12 %). The median age was 66 years (range 36–96 years). Patient characteristics are shown in Table 1.

**Table 1:** Patient characteristics

Patient characteristics:	
Number of patients:	163
Number of interventions:	206
Median age (range)	66 (36–96)
Tumor diagnosis	Number of patients (%)
Pancreatic carcinoma	60 (37)
Cholangiocellular carcinoma	34 (21)
Colorectal carcinoma	19 (12)
Hepatocellular carcinoma	9 (6)
Gastric cancer	8 (5)
Breast cancer	8 (5)
Neuroendocrine tumor of the digestive tract	6 (4)
Tumor of unknown origin	5 (3)
Ovarian carcinoma	3 (2)
Intraductal papillary mucinous neoplasm (IPMN)	2 (1)
Lymphoma	1 (1)
Other tumors	8 (5)
Laboratory parameters before ERC	Median (SD)
Bilirubin (mg/dl)	4.5 (6.83)
CRP (mg/l)	36.2 (62.11)

Among patients with pancreatic cancer, locally advanced disease was documented in 58 % of the cases, metastatic disease in 42 %.

A total of 206 ERCP interventions were performed due to suspected biliary obstruction. Biliary drainage with a stent was performed in 143 interventions (69.4 %). 

### Microbiological results

Bacterial colonization of the bile was common; overall, bacteria were detected in 128 samples (62 %). The most commonly isolated species were *Streptococcus* species (36 samples, 18 %), followed by *Enterococcus* species (35 samples, 17 %) and *Escherichia coli* and coagulase-negative *Staphylococcus* species (27 samples, 13 % respectively) (Table 2).

**Table 2:** Microbial isolation

Organism	Number of patients (%)
Bacteria	
*Streptococci*, alpha- and beta-hemolytic	36 (18)
*Enterococcus* spp.	35 (17)
*Escherichia coli*	27 (13)
Coagulase-negative *Staphylococcus*	27 (13)
*Klebsiella* spp.	14 (7)
*Clostridium*	1 (1)
Other bacteria	45 (22)
Sterile bile	50 (24)
Missing data	28 (14)
Multiresistant bacteria	
Vancomycin-resistant *Enterococcus faecium*	4 (2)
*E. coli* with extended spectrum beta-lactamase	4 (2)
Any multiresistant pathogen	14 (7)
Fungi	
*Candida albicans*	32 (16)
*Candida glabrata*	11 (5)
Missing data	76 (37)

Multiresistant gram-negative or gram-positive microorganisms, such as methicillin-resistant *Staphylococcus aureus* or vancomycin-resistant *Enterococcus*, were only rarely observed; these were detected in 14 samples (7 %).

Mycologic results were available for 130 interventions. *Candida albicans* was detected in 32 samples (16 %) and *Candida glabrata* was detected in 11 samples (5 %). Among patients with previous ERC, a significantly increased risk of a fungal biliary colonization was observed (10 of 73 patients without previous ERC vs. 33 of 62 patients with previous ERC, *p* <0.001).

Patients with proven fungobilia had a significantly longer period of antibiotic therapy before ERC intervention (median 6 days vs. 3 days; *p* = 0.020).

### Clinical outcome

In our series, patient outcome was associated with the microbial pathogens involved and the inflammatory state prior to ERC intervention.

**Fig. 1:**
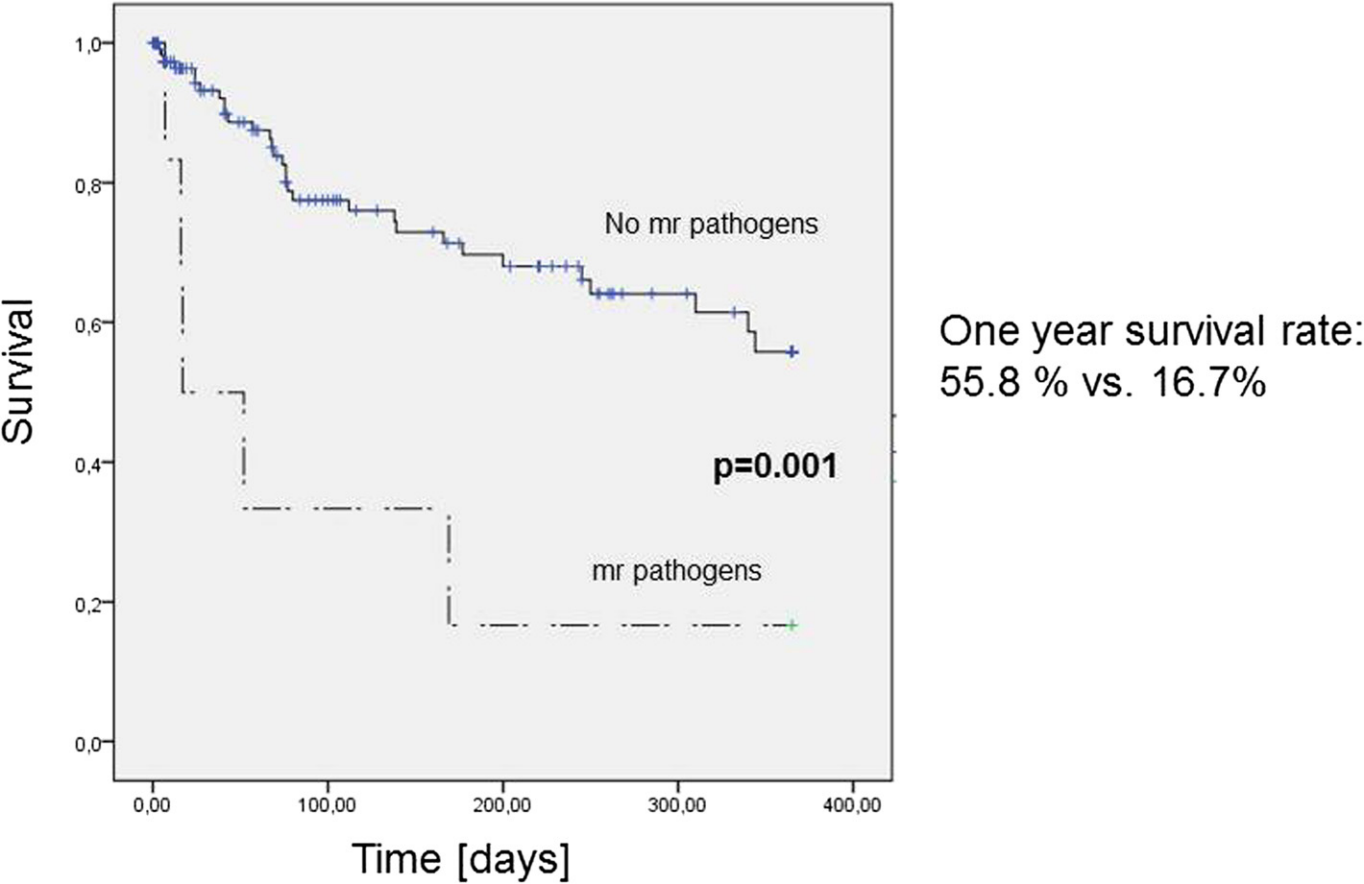
One-year survival in tumor patients according to the microbial isolation of multiresistent (mr) pathogens

Patients with a multiresistant pathogen isolated in bile during the first ERC intervention had a significantly worse one-year survival than patients without finding of a multiresistant species (One year survival rate 55.8 % vs. 16.7 %, *p* = 0.001) (Fig. 1).

**Fig. 2:**
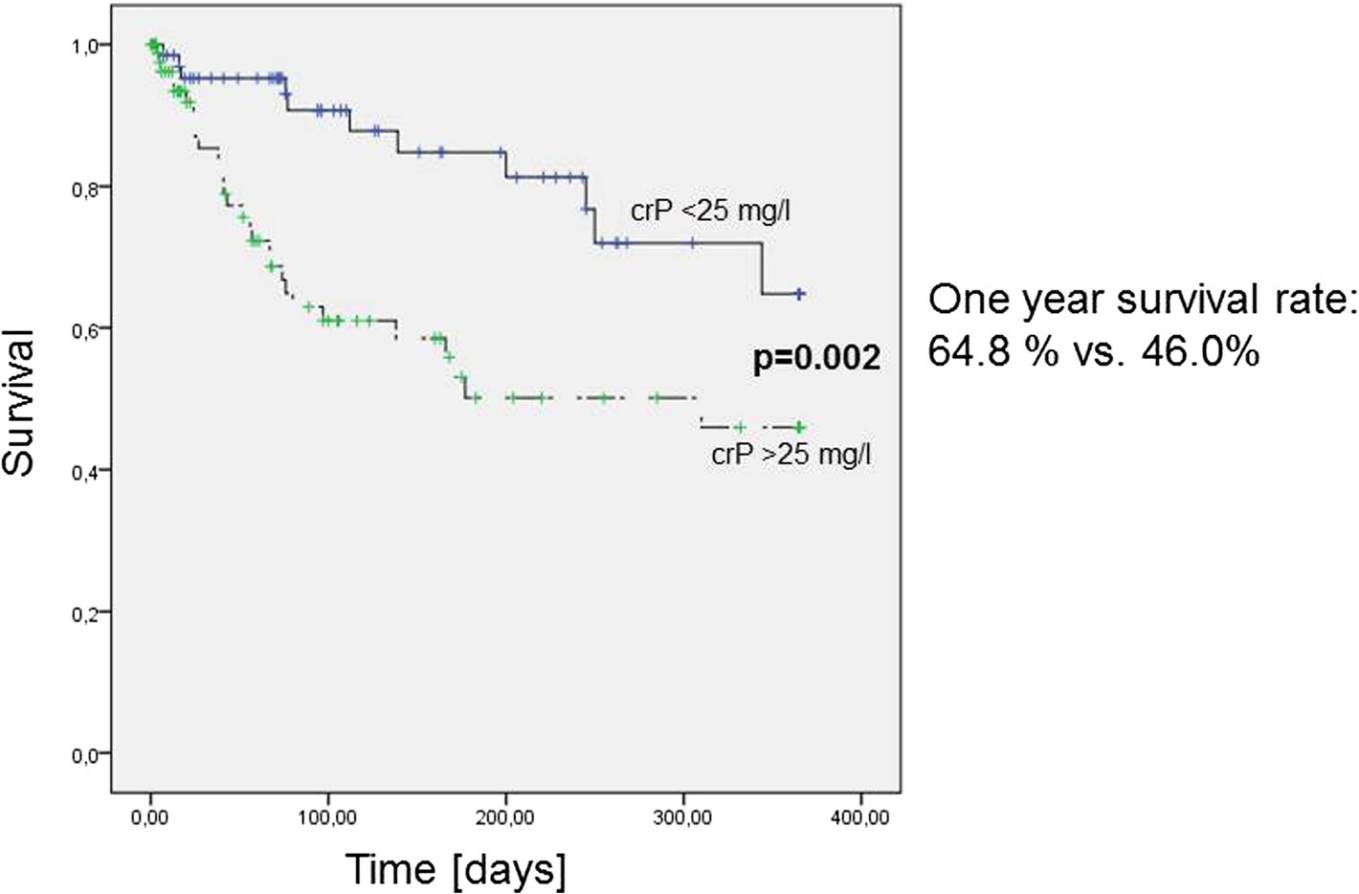
One-year survival in tumor patients according to preinterventional C-reactive protein level

Malignant obstruction, the resulting increased risk of cholangitis, and the advanced tumor diseases themselves can all contribute to a permanent state of inflammation. Distinguishing patients with a tumor-related inflammatory state from those with bacterial infection can be a clinical challenge. In our series, tumor patients presenting with increased levels of inflammatory markers at the first ERC intervention, defined by a C-reactive protein level higher than 25 mg/l (normal range, 0.5–5 mg/l), had a significantly worse one-year survival than patients without increased inflammation markers (One year survival rate 64.8 % vs. 46.0 %, *p* = 0.002) (Fig. 2).

Multivariate analysis including age (less or more than 65 years), the proof of any multiresistant pathogen, the proof of *Candida* species, placement of a stent, the primary tumor (pancreatic vs. non-pancreatic), a high bilirubin level (higher than 7 mg/dl) and a high CRP level (higher than 25 mg/l) confirmed the negative impact of a multiresistent pathogen (Hazard Ratio 8.77, *p* = 0.009) or a high CRP level (Hazard Ratio 2.98, *p* = 0.041) (Table 3).

**Table 3:** Multivariate analysis of all tumor patients

	p-value	Hazard Ratio	95 % Confidence Intervall
Placement of Stent (yes vs. no^a^)	0.245	2.12	[0.60;7.55]
Pancreatic primary tumor (yes vs. no^a^)	0.714	1.22	[0.43;3.50]
Bilirubin (≥7 mg/dl vs. <7 mg/dl^a^)	0.321	0.58	[0.20;1.69]
Age (≥65 years vs. <65 years^a^)	0.245	0.56	[0.21;1.49]
Proof of candida (yes vs. no^a^)	0.709	1.19	[0.47;3.04]
Proof of multiresistant pathogen (yes vs. no^a^)	0.009	8.77	[1.70;45.17]
CRP (≥25 mg/l vs. <25 mg/l^a^)	0.041	2.98	[1.05;8.50]

^a^reference

**Fig. 3:**
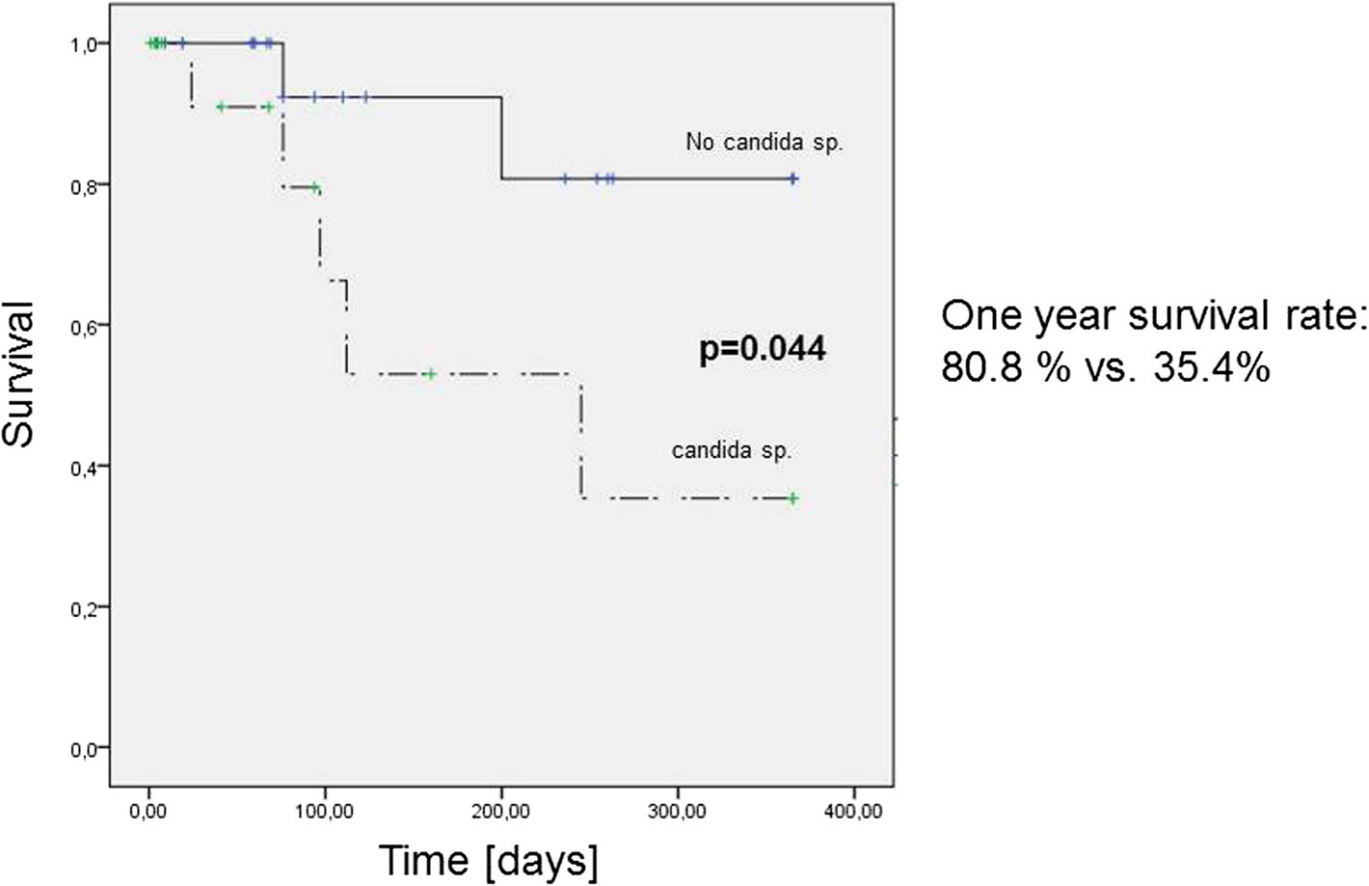
One-year survival in patients with pancreatic cancer according to the proof of *Candida* sp.

Among patients with pancreatic cancer, those in whom *Candida* species was found at the first ERC intervention had a significantly lower one-year survival than patients without findings of *Candida* in bile (One year survival rate 80.8 % vs. 35.4 %, *p* = 0.044) in univariate analysis (Fig. 3). Additionally, the pre-interventional bilirubin and C-reactive protein level at the first intervention did not differ significantly between these 2 groups.

Multivariate analysis including the proof of *Candida* species, placement of a stent, tumor stage (locally advanced vs. metastatic disease), a high bilirubin level (higher than 7 mg/dl) and a high CRP level (higher than 25 mg/l) confirmed the negative prognostic impact of the proof of *Candida* in the bile. (Hazard Ratio 23.07, *p* = 0.049) (Table 4).

**Table 4:** Multivariate analysis of pancreatic tumor patients

	p-value	Hazard Ratio	95 % Confidence Intervall
Placement of Stent (yes vs. no^a^)	0.830	0.80	[0.11;5.90]
Bilirubin (≥7 mg/dl vs. <7 mg/dl^a^)	0.982	0.98	[0.14;6.78]
Proof of candida (yes vs. no^a^)	0.049	23.07	[1.01;526.64]
CRP (≥25 mg/l vs. <25 mg/l^a^)	0.293	0.25	[0.02;3.31]
metastatic vs. locally adv. disease^a^	0.108	8.92	[0.62;128.38]

^a^reference

### Previous and further tumor-specific treatment

59 patients (36 %) presenting with advanced disease had received previous systemic therapy for their tumor disease, which had to be discontinued because of progressive jaundice. After the intervention, tumor-specific systemic treatment was (re)-initiated in 70 patients (43 %), after the malignant obstruction was alleviated due to successful drainage (Table 5).

**Table 5:** Previous and further treatment

Systemic therapy before the first ERC	Number of patients (%)
Yes	59 (36)
No	92 (56)
Unknown	12 (7)
Systemic therapy after ERC intervention	
Yes	70 (43)
No	36 (22)
Unknown	57 (35)

The prevalence of candida did not differ between pancreatic cancer patients with or without previous systemic chemotherapy (*p* = 0.661); in addition there was no statistical difference with regard to fungobilia in the rate of patients undergoing systemic treatment after successful ERC intervention (*p* = 0.631) or in the time till (re-)initiation of systemic chemotherapy (*p* = 0.161).

## Discussion

Data concerning the optimal management and outcome of patients with malignant bile duct obstruction are limited. For more than 20 years, ERC has been the method of choice to improve biliary drainage. Early intervention is desirable to prevent secondary complications such as liver failure or cholangitis. Additionally, an increase in the performance score can often be observed after relief of jaundice.

In our series, the detected bacterial species were common pathogens, which could be controlled with standard antibiotic treatment. In contrast to other cohorts (e.g. patients who underwent liver transplantation [11–13]) , multiresistant bacteria were only rarely observed, but these patients had a significantly worse outcome.

Among all cancer patients, those with increased C-reactive protein levels had a significantly worse outcome than those with low levels of inflammatory markers. In concordance to our data Iwasaki et al. showed that an inflammation-based prognostic score (mGPS, modified Glasgow Prognostic Score) including C-reactive protein and hypalbuminemia is a significant predictor of postoperative survival in patients undergoing palliative surgery for malignant biliary obstruction [14]. It is unknown whether these increased markers are a sign of a more severe bacterial cholangitis or the result of a tumor-related inflammatory status due to a more advanced disease. Other more specific markers like procalcitonin could be useful in distinguishing between a bacterial infection and a tumor-related inflammatory state [15].

The prognostic role of fungi detected in the bile of patients with malignant biliary obstruction is not yet well defined. In our series, *Candida* species was detected in approximately 25 % of all pancreatic cancer patients; if *Candida* species was detected in the bile, the patients had a significantly worse one-year survival than those in whom *Candida* was not detected. The negative prognostic impact was confirmed in multivariate analysis. The reason for this effect is unknown; proof of *Candida* was neither associated with previous immunosuppressive therapy (cytotoxic chemotherapy) nor with increased levels of inflammatory markers or cholestasis parameters. These findings were in contrast to those for the rest of the patients who had malignant jaundice. No significant difference was noted in the rate of *Candida* detection between the groups with locally advanced and metastatic cancer. *Candida* might therefore have a negative impact on outcome in patients with pancreatic cancer and malignant biliary obstruction. A randomized trial with antimycotic treatment in this cohort might help in defining the pathognomonic role of this species in the bile.

With regard to previous and post-interventional tumor-specific therapy, more than one-third of our patients had received previous tumor-specific medical therapy, which had to be discontinued because of tumor progression with obstructive jaundice. After the ERC intervention, a start or re-initiation of tumor-specific therapy is documented to have occurred in 43 % of patients, highlighting the widening oncological therapeutic options once the biliary drainage is improved.

## Conclusions

In summary, endoscopic intervention for cancer patients is a feasible and effective method of improving biliary drainage. It should be performed as soon as malignant obstruction is diagnosed in order to permit the start or continuation of tumor-specific therapy. The bacterial species found in the bile of cancer patients were mostly common pathogens, which could be controlled with standard antibiotic treatment.

Our data showed a negative impact of *Candida* on one-year survival in patients with pancreatic cancer. Whether an antimycotic treatment could overcome this negative outcome is unknown. Thus, the role of *Candida* in the bile should be explored in a randomized trial.

## Abbreviations

CRP: C-reactive protein
ERC(P): Endoscopic retrograde cholangio(pancreato) graphy
HCC: Hepatocellular carcinoma
mGPS: modified Glasgow Prognostic Score
